# Machine learning cluster analysis identifies increased 12-month mortality risk in transcatheter aortic valve replacement recipients

**DOI:** 10.3389/fcvm.2025.1444658

**Published:** 2025-02-05

**Authors:** Thomas Meredith, Farhan Mohammed, Amy Pomeroy, Sebastiano Barbieri, Erik Meijering, Louisa Jorm, David Roy, Jason Kovacic, Michael Feneley, Christopher Hayward, David Muller, Mayooran Namasivayam

**Affiliations:** ^1^ Department of Cardiology, St Vincent's Hospital, Sydney, NSW, Australia; ^2^ Heart Valve Disease & Artificial Intelligence Laboratory, Victor Chang Cardiac Research Institute, Sydney, NSW, Australia; ^3^ Faculty of Medicine and Health, University of New South Wales, Sydney, NSW, Australia; ^4^ Centre for Big Data in Health Research, University of New South Wales, Sydney, NSW, Australia; ^5^ Queensland Digital Health Centre, University of Queensland, Brisbane, QLD, Australia; ^6^ School of Computer Science and Engineering, University of New South Wales, Sydney, NSW, Australia; ^7^ Cardiovascular Research Institute, Icahn School of Medicine at Mount Sinai, New York, NY, United States

**Keywords:** aortic stenosis, machine learning, outcomes, cardiac imaging, transcatheter aortic valve replacement

## Abstract

**Background:**

Long-term mortality risk is seldom re-assessed in contemporary clinical practice following successful transcatheter aortic valve implantation (TAVR). Unsupervised machine learning permits pattern discovery within complex multidimensional patient data and may facilitate recognition of groups requiring closer post-TAVR surveillance.

**Methods:**

We analysed and differentiated routinely collected demographic, biochemical, and cardiac imaging data into distinct clusters using unsupervised machine learning. k-means clustering was performed on data from 200 patients who underwent TAVR for severe aortic stenosis (AS). Input features were ranked according to their influence on cluster assignment. Survival analyses were performed with Kaplan–Meier and Cox proportional hazards models. Nested cox models were used to identify any incremental prognostic benefit cluster assignment achieved beyond conventional risk scores.

**Results:**

Analysis identified two distinct clusters. Compared to Cluster 1, Cluster 2 demonstrated significantly worse all-cause mortality at 12 months (HR 6.3, *p* < 0.01), and was characterised by more advanced cardiac remodelling with worse indices of multi-chamber cardiac function, as quantified by strain imaging. Cluster assignment demonstrated greater predictive power for 12-month mortality as compared with conventional risk and frailty calculators.

**Conclusion:**

k-means clustering identified two prognostically distinct phenogroups of patients who had undergone TAVR with better discriminatory power than conventional risk and frailty calculators. Our results highlight the utility of machine learning applications for clinical risk prediction and scope to improve patient surveillance.

## 1 Introduction

Aortic stenosis (AS), one of the most common degenerative valvular conditions, is increasing in prevalence (1). Transcatheter aortic valve replacement (TAVR) is now the most common treatment modality for severe symptomatic aortic stenosis (AS), while open surgery is now generally reserved for younger patients with longer life-expectancy (2). Although TAVR was initially only indicated for patients at prohibitive perioperative open surgical mortality risk, there is now robust evidence supporting its non-inferiority, and even superiority, across the entire peri-operative risk spectrum. As such, contemporary patients undergoing TAVR represent a diverse mix of risk and frailty profiles. Prediction of *peri-operative* risk is usually performed with one or more well established tools, such as the Society of Thoracic Surgery (STS) predicted risk of mortality (PROM) calculator, the EuroSCORE II, and frailty tools such as the Rockwood Clinical Frailty Score (3). Multiple TAVR-specific risk scores have been also developed (4–7). However, outside of clinical trials and registries, mortality risk is seldom re-assessed following successful TAVR and it is presently unclear how to best quantify residual prognostic risk for the overwhelming majority of patients who survive their procedure. Moreover, follow-up recommendations are largely based on surveillance of echocardiographic valve function parameters—namely trans-prosthesis gradients and calculated effective orifice/valve area (EOA), irrespective of clinical risk or frailty scores (8). A recent analysis of the Nationwide Readmissions Database by Elkaryoni et al. revealed that nearly a third of patients experience at least one rehospitalization in the first 90 days following successful TAVR (9).

Unsupervised machine learning (ML), a field of artificial intelligence (AI), enables the discovery of important patterns within complex multidimensional data, such as cardiac imaging data (10). Given the current paradigm of risk stratification based on the limited selection of key validated variables, the use of ML could enable risk prediction with much greater accuracy by incorporating much more expansive and complex data. Specifically, we postulated that prognostically distinct phenogroups might be discoverable when comprehensive, multidimensional patient data were subjected to unsupervised cluster analysis. We sought to investigate whether cluster analysis of data including features of patient demography, biochemistry, CT valve geometry and post-implant echocardiography would identify groups with meaningful differences in survival, and potentially lay the foundation for ML guided post-procedural patient surveillance (Central Illustration).

**Central Illustration:**
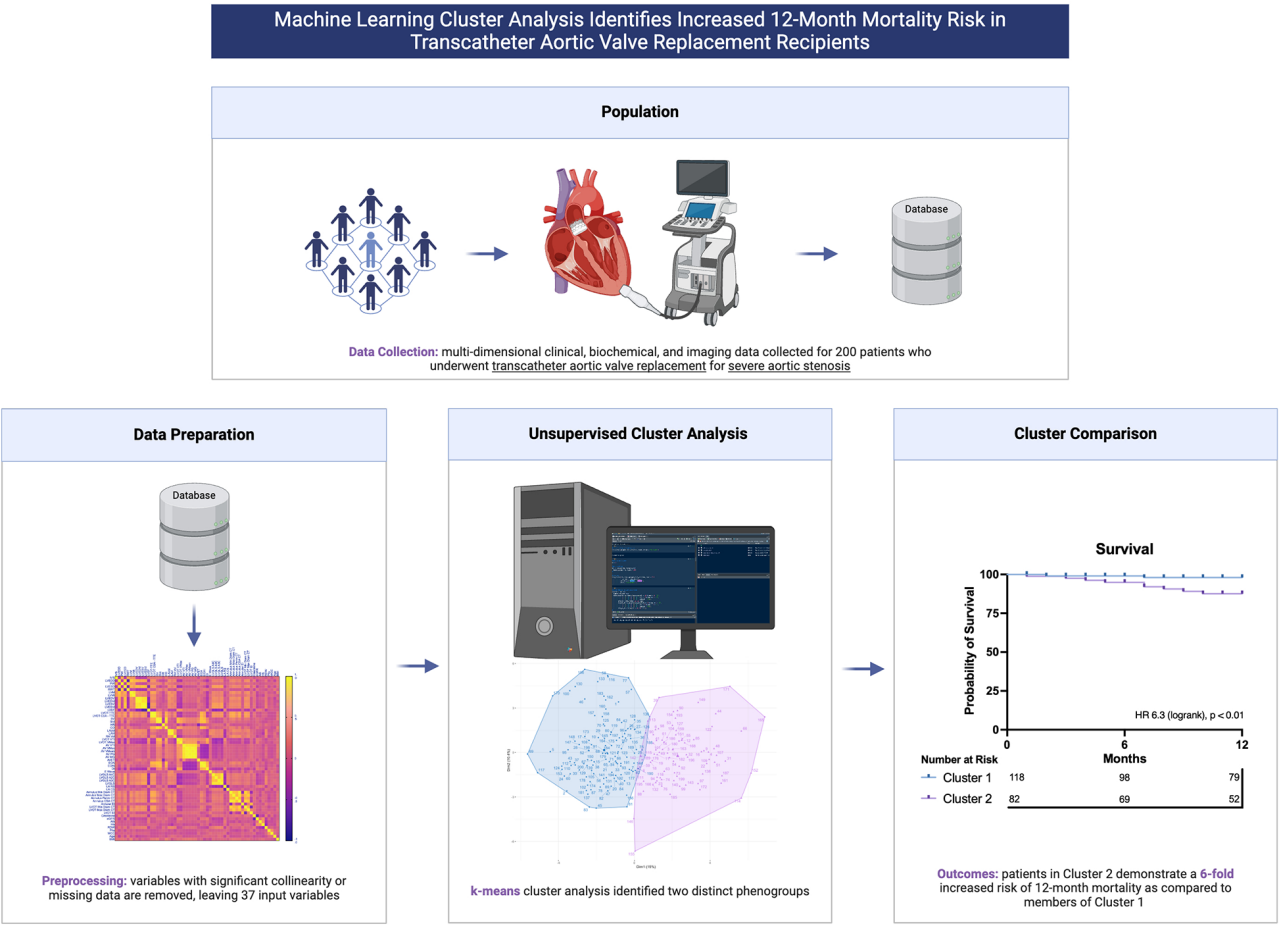
Machine learning cluster analysis of multi-dimensional patient data identifies prognostically distinct phenogroups of patients who have undergone transcatheter aortic valve replacement.

## 2 Methods

### 2.1 Case selection

Our institutional database was screened for patients who had undergone TAVR with available post-procedural echocardiograms of suitable image quality for analysis. At minimum, post-procedural studies needed to include apical 2-, 3- and 4-chamber views to facilitate chamber quantification and speckle tracking. The database was screened from 2018 to June 2023. Cases were excluded if imaging was not performed on-site, or the indication for TAVR was primary aortic regurgitation or valve-in-valve procedures. Baseline demographic, comorbidity and procedural data were abstracted from hospital medical records and clinic letters. Follow-up censoring occurred at last known interaction with state-based health services. The study was approved by the local institutional ethics board (St Vincent's Hospital Human Research Ethics Committee ETH2021/11608).

### 2.2 Imaging analysis

Raw echocardiographic images were de-identified and re-analysed by an accredited Cardiologist or Cardiac Sonographer at the Heart Valve Disease and Artificial Intelligence Laboratory at the Victor Chang Cardiac Research Institute, Sydney. Conventional chamber measurements were performed according to contemporary ASE guidelines (11). Left ventricular global longitudinal strain (GLS) analysis was performed using TomTec Arena software (TOMTEC Imaging Systems, Germany), using apical 2-, 3- and 4-chamber views. Studies were only included for analysis if the left ventricular endocardium could be visualized and endocardial tracking was accurate in these views throughout the cardiac cycle. Routine TAVR planning CT scans were analysed using vendor-neutral processing software (3mensio, Pie Imaging) to determine the dimensions and area of the left ventricular outflow tract (LVOT) and the aortic annulus. The LVOT was planimetered 3 mm below the aortic annulus. The eccentricity index was calculated for the annulus and LVOT, as 1- d_min_/d_max_, where 0 represents a perfect circle, as described by Leipsic et al. (12).

### 2.3 Data preparation

A total of 55 continuous variables, comprising a combination of demographic, biochemical, echocardiographic and CT measurements, were included following removal of variables with >15% missing data. We used all available continuous variables from our database, to mitigate selection bias caused by only selecting features of putative importance. A Pearson's correlation heatmap was constructed to assess for collinearity (Supplementary Figure S1). Variables which demonstrated significant collinearity (Pearson correlation coefficient >0.8) were excluded (Supplementary Table S1), retaining the most general variable for multi-constituent variables, or variable counterparts indexed to body surface area (BSA). This left 37 variables, listed in Table 1. For the purposes of unsupervised clustering, missing data (for the included variables below the 15% missingness threshold) were imputed using multiple imputation by chained equations (13). All variables were normalized to have a mean of zero and a standard deviation of one. Following cluster assignment, original non-imputed data were used for cluster comparison and survival assessment.

**Table 1:** Variables included in k-means clustering algorithm, following removal of variables with significant collinearity.

Feature class	Features
Clinical & biochemical	Age BMI Haemoglobin Serum albumin Red cell distribution width Serum creatinine eGFR White cell count Platelet count
Echocardiography	Interventricular Septal Width (IVS) Posterior Wall Width (PW) LV end diastolic diameter (LVEDD) LV end diastolic volume index (LVEDVi) E Wave Relative Wall Thickness (RWT) LV Mass Index (LVMi) LVOT diameter LVOT VTI Stroke Volume Index (SVi) Heart Rate (HR) Cardiac Output (CO) LA Volume Index (LAVi) RA Volume Mean TAVR Gradient Aortic Valve Ejection Time (AVET) Indexed TAVR Effective Orifice Area Dimensionless Index (DI) LVEF LV GLS LA reservoir strain LA conduit strain
Computed tomography	Annular perimeter Annular cross-sectional area Annular eccentricity Index LVOT minimum diameter LVOT maximum diameter LVOT eccentricity Index

### 2.4 Cluster allocation

K-means clustering was performed, which has been previously employed in similar phenotyping studies (14, 15). In contrast to other clustering approaches, such as hierarchical clustering, we favoured k-means methodology for computationally efficiency, simplicity, and interpretability (16). The optimal number of clusters (k) was determined by iteratively including sequentially greater cluster numbers in the algorithm and inspecting the respective silhouette scores, which measure intra-cluster similarity and inter-cluster dissimilarity. Following identification of optimal k, clustering was performed using random centroid initializations, the most robust of which were selected for cluster allocation. To evaluate the robustness of the identified clusters, we performed a subsampling-based stability analysis. Subsamples comprising 80% of the dataset were generated 100 times, and k-means clustering was applied to each subset (at optimal k). Cluster assignments were compared to the full dataset using the Adjusted Rand Index (ARI), which quantifies similarity while adjusting for chance. The mean and standard deviation of the ARI across subsamples were calculated to assess the consistency and stability of the clusters.

### 2.5 Cluster characteristic analysis

Characteristics between clusters were compared using Wilcoxon rank sum, Fisher's exact and Chi-squared tests, as required by variable type and distribution. Continuous variables are presented as median and interquartile range and categorical variables as counts (n) and percentages. For the purposes of identifying potentially predictive features of cluster allocation, we performed two analyses. Firstly, we performed simple analysis of variance between each feature and then ranked each feature by F statistic value. We also trained a logistic regression model to predict cluster allocation based on the pre-processed k-means input features (15). Logistic regression model performance was determined using accuracy, sensitivity, specificity, and area under receiver-operator characteristic (ROC-AUC) curve analysis. Kaplan–Meier curves were plotted for all-cause mortality and compared with the logrank test. Cox proportional hazards analyses were performed to assess the influence of cluster assignment on mortality. To assess any incremental prognostic benefit cluster assignment achieved beyond conventional risk scores, we created four nested cox models, and compared performance with Harrell's C-Index and Global *χ*^2^ (likelihood ratio) scores. Analyses were performed using R (R Foundation for Statistical Computing, Vienna, Austria) and graphics generated with GraphPad Prism v10. The source code will be made available on GitHub upon publication of this paper.

This study was conducted according to the Transparent Reporting of a Multivariable Prediction Model for Individual Prognosis or Diagnosis (TRIPOD) statement (Supplementary Table S2) (17).

## 3 Results

### 3.1 Baseline characteristics

From 2018 to 2023, 840 patients underwent TAVR at our institution. Following exclusion of patient with imaging of inadequate quality or not performed at our institution, the cohort comprised 200 patients. Baseline characteristics for the 200 included patients are outlined in Table 2. The median age was 82 years, and 112 participants were male (56%). 170 patients (85%) were implanted with a self-expanding prosthesis. The median STS score was 4.2% (IQR 2.5–6.5) and 21% of patients had a Clinical Frailty Score (CFS) of 5 or more, consistent with significant frailty. With respect to post-procedural cardiac function, the median LVEF was 62%, LV GLS 19.6%, RV GLS 25%. The median post-implant gradient and EOA were 7.3 mmHg (IQR 5.9–10.3) and 1.76 cm^2^ (IQR 1.46–2.14), respectively. Most participants (81%) had mild or less paravalvular incompetence, as quantified by the circumferential extent of detectable incompetence on parasternal short axis echocardiography.

**Table 2:** Cohort and cluster characteristics.

Characteristic	Overall, *N* = 200^a^	Cluster 1, *N* = 118^a^	Cluster 2, *N* = 82^a^	*p*-value^b^
Clinical & Demographic Data				
Age	82 (77, 86)	83 (78, 86)	82 (76, 87)	0.3
Male	112 (56%)	48 (41%)	64 (78%)	**<0**.**001**
BMI	26.3 (23.5, 30.1)	27.0 (23.5, 30.1)	26.0 (23.9, 30.0)	0.8
Hypertension	140 (70%)	93 (79%)	47 (57%)	**0**.**001**
CAD	101 (51%)	49 (42%)	52 (63%)	**0**.**002**
Atrial Fib/Flutter	62 (31%)	23 (19%)	39 (48%)	**<0**.**001**
Diabetes	49 (25%)	27 (23%)	22 (27%)	0.5
Hyperlipidaemia	150 (81%)	91 (81%)	59 (81%)	>0.9
Previous Stroke/TIA	21 (11%)	8 (6.8%)	13 (16%)	**0**.**040**
Active Smoker	4 (2.0%)	3 (2.5%)	1 (1.2%)	0.6
Severe Pulmonary Disease	32 (17%)	15 (14%)	17 (22%)	0.14
OSA	18 (9.5%)	10 (9.0%)	8 (10%)	0.8
Cognitive Impairment	11 (5.9%)	8 (7.2%)	3 (3.9%)	0.5
CKD	76 (38%)	33 (28%)	43 (52%)	**<0**.**001**
STS Score	4.2 (2.5, 6.5)	4.2 (2.4, 6.5)	4.2 (2.7, 6.5)	>0.9
Clinical Frailty Score >4	34 (21%)	17 (17%)	17 (26%)	0.2
NYHA Score >2	41 (21%)	17 (14%)	24 (30%)	**0**.**009**
Biochemical Data				
Hb	126 (114, 137)	126 (119, 136)	123 (110, 137)	0.2
Platelets	202 (162, 247)	213 (173, 253)	196 (156, 226)	**0**.**033**
WCC	6.70 (5.60, 7.70)	6.80 (5.80, 7.60)	6.65 (5.40, 7.90)	0.8
Serum albumin	36.0 (33.0, 39.0)	36.0 (34.0, 39.0)	34.0 (32.3, 37.8)	**0**.**022**
Serum creatinine	91 (74, 115)	79 (67, 102)	110 (84, 132)	**<0**.**001**
eGFR	61 (46, 77)	65 (50, 80)	52 (42, 72)	**<0**.**001**
Device Data				
TAVR Device				**0**.**012**
Sapien 3	24 (12%)	11 (9.3%)	13 (16%)	
Sapien 3 Ultra	6 (3.0%)	2 (1.7%)	4 (4.9%)	
Evolut-R	125 (63%)	70 (59%)	55 (67%)	
Evolut-Pro	38 (19%)	28 (24%)	10 (12%)	
Portico	7 (3.5%)	7 (5.9%)	0 (0%)	
Post-Procedural Echocardiography Data				
HR	73 (65, 82)	72 (65, 82)	75 (68, 82)	0.14
IVS Width (mm)	12.80 (11.44, 13.98)	12.89 (11.73, 14.21)	12.43 (11.31, 13.83)	0.14
PW Width (mm)	12.12 (11.05, 13.18)	12.34 (11.30, 13.21)	11.93 (10.87, 12.93)	0.3
LVEDD (mm)	44 (40, 49)	41 (38, 45)	49 (44, 54)	**<0**.**001**
LVEDV (mls)	76 (59, 97)	62 (51, 78)	102 (85, 124)	**<0**.**001**
LVEDVi (mls/m^2^)	41 (33, 53)	36 (30, 42)	53 (45, 64)	**<0**.**001**
LVESD (mm)	28 (25, 33)	26 (23, 29)	35 (29, 41)	**<0**.**001**
LVESV (mls)	28 (20, 43)	22 (16, 28)	46 (33, 65)	**<0**.**001**
LVESVi (ml/m^2^)	16 (11, 24)	12 (9, 16)	25 (18, 36)	**<0**.**001**
LV Mass Index (g/m^2^)	113 (95, 130)	106 (91, 120)	124 (107, 143)	**<0**.**001**
LVEF %	62 (52, 68)	67 (61, 71)	53 (43, 62)	**<0**.**001**
LV GLS %	−19.6 (−21.8, −16.9)	−21.4 (−22.8, −19.5)	−17.2 (−19.0, −13.8)	**<0**.**001**
SVi (mls/m^2^)	35 (29, 44)	38 (32, 45)	30 (26, 36)	**<0**.**001**
Cardiac Output (L/min)	4.70 (3.73, 5.68)	5.12 (3.91, 6.14)	4.38 (3.59, 4.86)	**<0**.**001**
E (cm/s)	0.98 (0.81, 1.21)	0.97 (0.80, 1.17)	1.01 (0.85, 1.27)	0.5
e′	0.069 (0.059, 0.082)	0.067 (0.059, 0.079)	0.071 (0.060, 0.095)	0.15
TAVR Mean Gradient (mmHg)	7.3 (5.9, 10.3)	7.8 (6.1, 11.1)	7.0 (5.8, 9.8)	**0**.**048**
TAVR Peak Velocity (cm/s)	1.87 (1.68, 2.19)	1.93 (1.70, 2.31)	1.79 (1.60, 2.12)	0.051
DI	0.64 (0.51, 0.75)	0.70 (0.57, 0.80)	0.53 (0.46, 0.64)	**<0**.**001**
EOA (cm^2^)	1.76 (1.46, 2.14)	1.83 (1.51, 2.21)	1.66 (1.38, 2.05)	0.083
EOAi (cm^2^/m^2^)	0.99 (0.77, 1.15)	1.04 (0.83, 1.20)	0.88 (0.73, 1.07)	**0**.**006**
Patient-Prosthesis Mismatch	43 (22%)	18 (15%)	25 (30%)	**0**.**010**
RVSP (mmHg)	28 (23, 34)	28 (23, 34)	29 (23, 37)	0.5
RA Volume (mls)	53 (38, 73)	43 (33, 54)	72 (56, 85)	**<0**.**001**
LA Volume (mls)	85 (69, 103)	74 (62, 90)	100 (85, 119)	**<0**.**001**
LAVi (mls/m^2^)	45 (37, 57)	41 (36, 52)	52 (44, 63)	**<0**.**001**
LA Reservoir Strain %	19 (14, 25)	21 (17, 28)	15 (11, 21)	**<0**.**001**
LA Contractile Strain %	−11 (−15, −7)	−11 (−17, −8)	−8 (−11, −6)	**<0**.**001**
RV Strain %	−25 (−29, −21)	−27 (−31, −22)	−21 (−26, −18)	**<0**.**001**
Mitral Regurgitation				**<0**.**001**
None	39 (20%)	33 (28%)	6 (7.3%)	
Trivial/Mild	139 (70%)	77 (65%)	62 (76%)	
Moderate	21 (11%)	8 (6.8%)	13 (16%)	
Severe	1 (0.5%)	0 (0%)	1 (1.2%)	
Tricuspid Regurgitation				0.7
None	61 (31%)	37 (31%)	24 (29%)	
Trivial/Mild	112 (56%)	68 (58%)	44 (54%)	
Moderate	23 (12%)	11 (9.3%)	12 (15%)	
Severe	4 (2.0%)	2 (1.7%)	2 (2.4%)	
Circumferential Extent of PVL (mm)	8 (6, 13)	8 (5, 14)	9 (6, 13)	0.8
PVL Severity				0.7
None	101 (51%)	63 (53%)	38 (46%)	
Mild	60 (30%)	33 (28%)	27 (33%)	
Moderate	36 (18%)	21 (18%)	15 (18%)	
Severe	3 (1.5%)	1 (0.8%)	2 (2.4%)	
CT Data				
Annulus Eccentricity Index	0.22 (0.16, 0.25)	0.23 (0.17, 0.25)	0.21 (0.16, 0.24)	0.3
LVOT Eccentricity Index	0.25 (0.20, 0.31)	0.26 (0.22, 0.32)	0.24 (0.19, 0.28)	**0**.**029**
LVOT CSA (cm^2^)	4.55 (3.91, 5.29)	4.09 (3.54, 4.58)	5.32 (4.79, 5.78)	**<0**.**001**
Annulus CSA (cm^2^)	4.61 (4.10, 5.29)	4.26 (3.80, 4.75)	5.30 (4.64, 5.78)	**<0**.**001**
STJ CSA (cm^2^)	6.47 (5.35, 7.35)	5.90 (4.97, 7.07)	7.00 (6.16, 7.62)	**<0**.**001**

Bold indicates *p* < 0.05.

^a^ Median (IQR), *n* (%).

^b^ Wilcoxon Rank sum test; Pearson's Chi-squared test; Fisher's exact test.

### 3.2 K-means clustering

**Figure 1:**
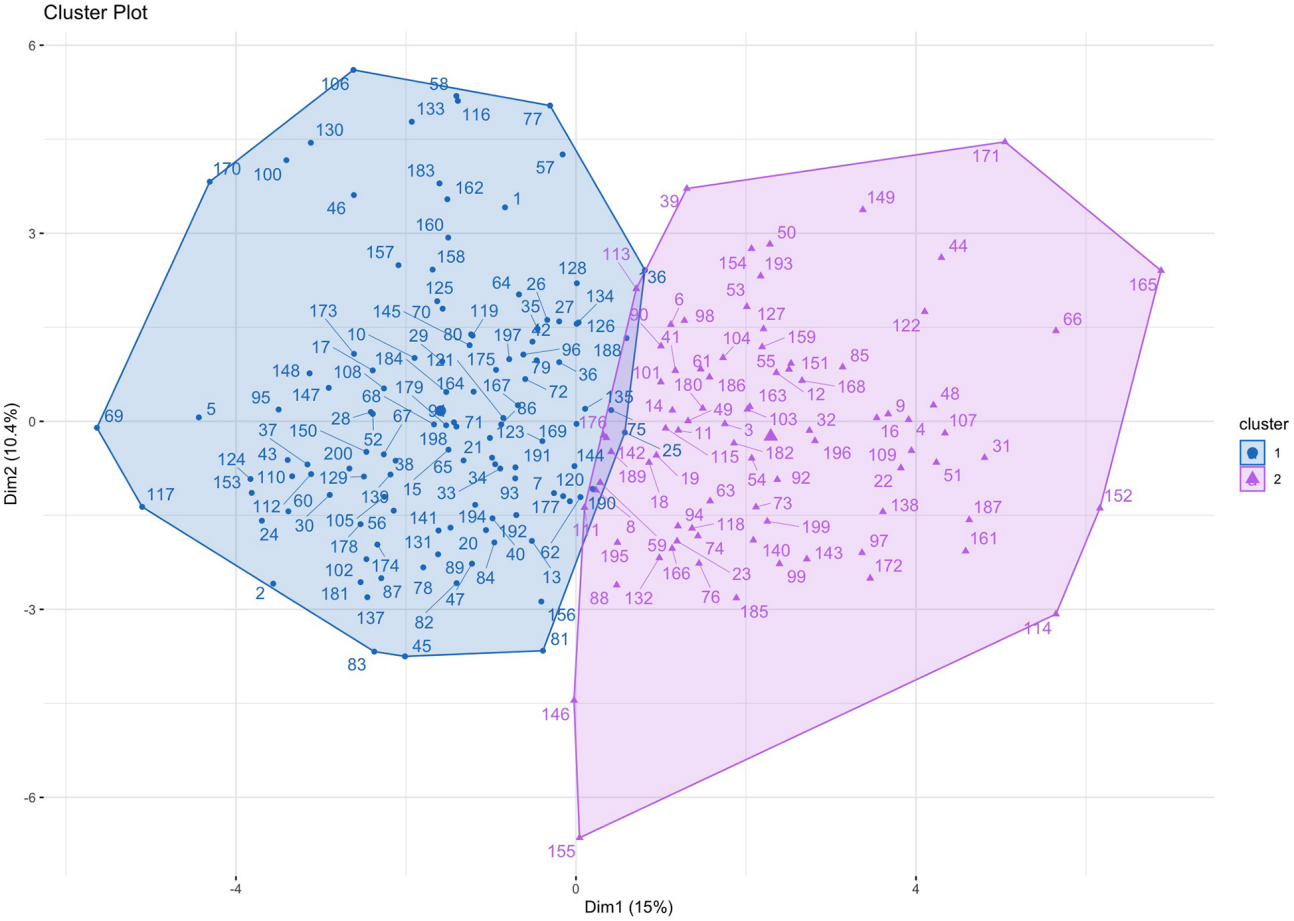
Cluster plot. Patients are plotted according to the first two principal components of their data, labelled Dimension 1 and Dimension 2.

Silhouette scores were calculated for models containing up to 10 clusters. The use of 2 clusters demonstrated the best (highest) silhouette score (Supplementary Figure S2). Principal component analysis was performed to visualise the clusters (Figure 1). The mean ARI across subsamples was 0.87 (±0.09), reflecting highly robust clustering. Characteristics for patients assigned to each of the two clusters are outlined in Table 2. Cluster 1 was larger than Cluster 2 and included proportionally more females. Age, baseline STS score and Clinical Frailty Score (CFS) were not significantly different between clusters. In Cluster 2 there was a higher prevalence of atrial fibrillation, coronary artery disease, stroke and chronic kidney disease, while hypertension was more prevalent in Cluster 1. Patients in Cluster 2 had lower platelet levels, serum albumin and eGFR, and higher serum creatinine levels. Patients in Cluster 2 had worse indices of multi-chamber cardiac function, specifically a lower LVEF, LV GLS, LA contractile and reservoir strain and RV strain. Cluster 2 patients also had larger left ventricular chamber dimensions, greater left ventricular mass (indexed to BSA), as well as greater left and right atrial volumes and mitral valve regurgitation. Patients in Cluster 2 also demonstrated large left ventricular outflow tract and annular geometry. With respect to prosthesis function, there was a small difference in trans-prosthetic gradients of borderline significance, but EOAs were similar between clusters. However, patients in Cluster 2 demonstrated smaller *indexed* EOAs consistent with greater prevalence of patient prosthesis mismatch and lower dimensionless indices.

### 3.3 Survival

**Figure 2:**
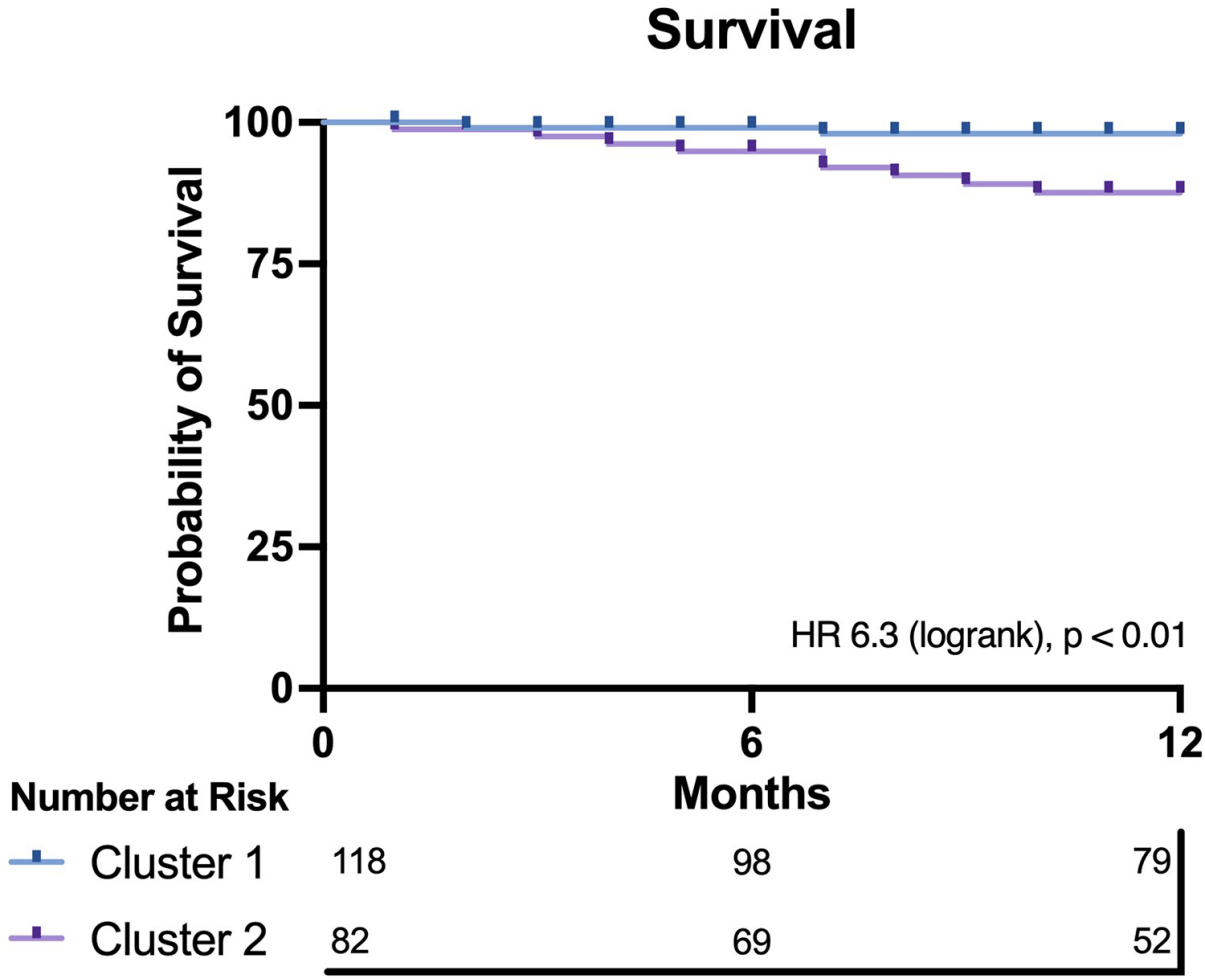
Kaplan–meier plot for both clusters. Cluster 2 demonstrates significantly worse 12-month survival compared with Cluster 1.

**Figure 3:**
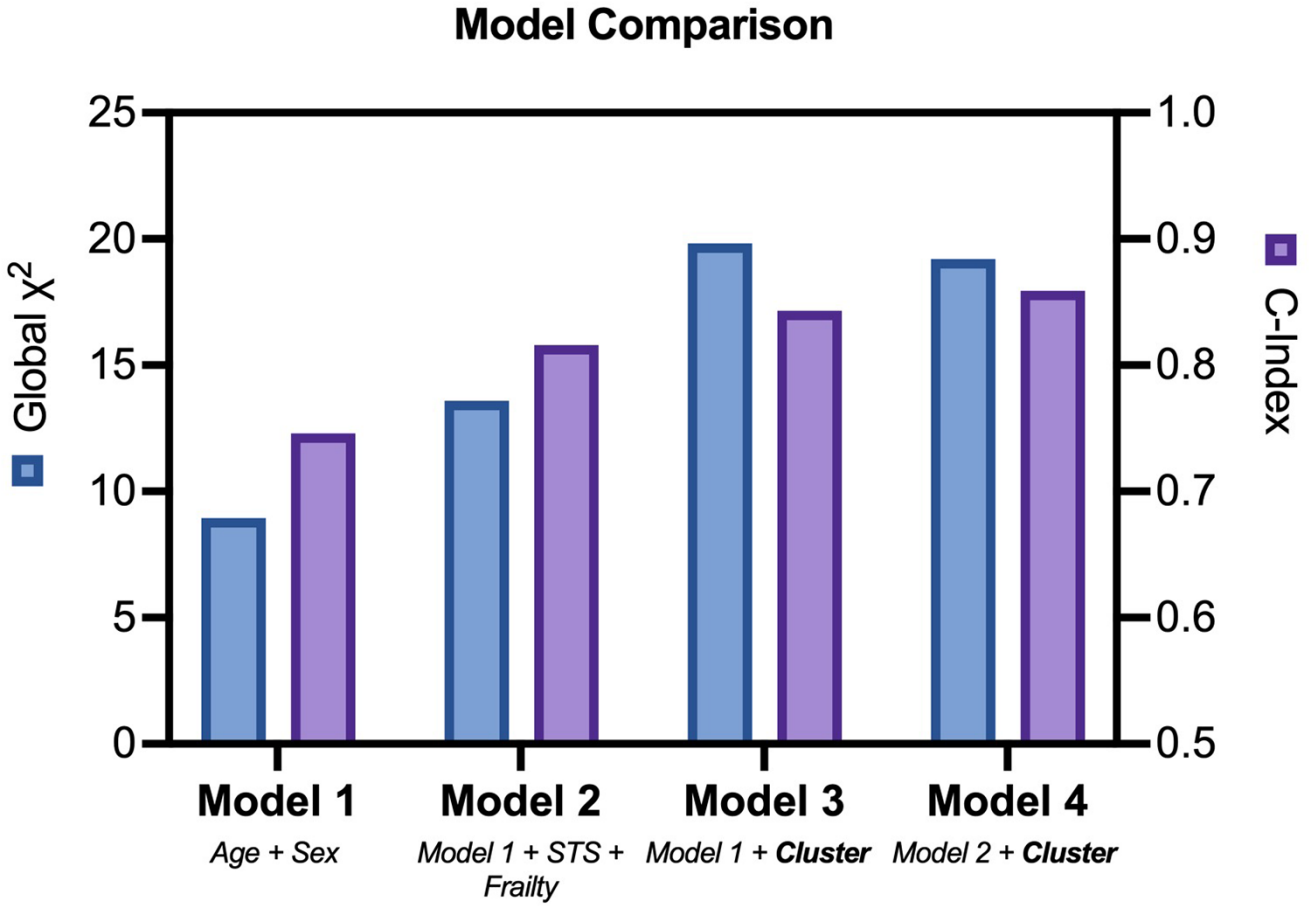
Nested cox models. Comparison of nested cox hazards models including clinical variables, risk and frailty scores, with or without cluster assignment.

Outcome data were available for all participants. The median follow-up time was 16.5 months, and longest follow up was 71 months. Patients in Cluster 2 demonstrated significantly worse all-cause mortality at a 12-month landmark analysis (logrank HR 6.3, 95% CI: 1.9–20.9, *p* < 0.01; Figure 2). A *post-hoc* power analysis was performed, revealing sufficient power to detect a 12-month mortality difference between clusters (85.1%, alpha 0.05). There was no signal of very early mortality at the one-month mark (*p* = 0.99). At longest follow-up, survival curves remained divergent but statistical significance was not met due to reduced patient numbers (Supplementary Figure S3). Cox proportional hazards modelling for mortality demonstrated that allocation to Cluster 2 was associated with a hazard ratio of 6.3 for all-cause mortality at 12 months (*p* = 0.018, 95% CI: 1.36–29.26). Nested models are illustrated in Figure 3. Adding cluster allocation to models containing simple clinical variables (age and sex, Model 1), as well as STS and Clinical Frailty scores (Model 2), significantly improved model performance (Model 3; Global *χ*^2^ = 19.82, C-Index 0.84, *p* < 0.01). Model 3, containing simple clinical variables and cluster assignment, was not significantly enriched with the addition of contemporary risk scores (STS and CFS, Model 4, Global *χ*^2^ = 19.21, C-Index 0.856, *p* < 0.002).

### 3.4 Feature importance

**Figure 4:**
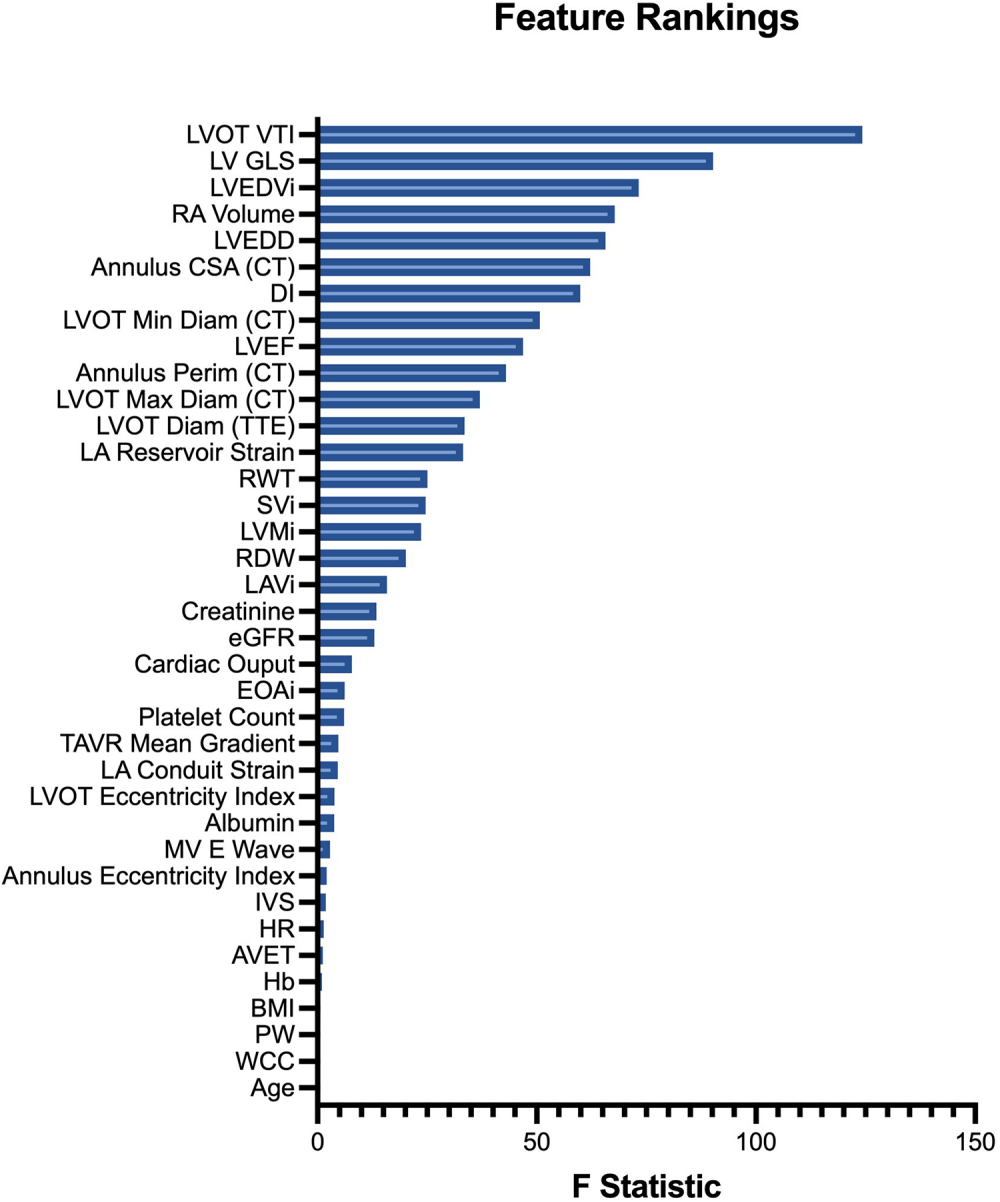
Input feature rankings. Features (variables) are ranked in descending order of their ANOVA F statistic, reflecting their relative importance to cluster assignment.

Feature rankings were similar between analysis of variance (Figure 4) and logistic regression methods (Supplementary Figures S4, S5). The four most influential variables with respect to cluster discrimination were the LVOT VTI, LV GLS, LVEDVi and RA volume. The four least influential variables were age, white cell count (WCC), posterior wall width and BMI.

## 4 Discussion

Unsupervised machine learning identified two prognostically distinct phenogroups within our population of patients undergoing TAVR. The prognostically less favourable cluster, which demonstrated a 6-fold increased risk of 12-month mortality, included patients with evidence of more advanced cardiac remodelling (larger left ventricular and atrial dimensions with greater left ventricular mass) and poorer indices of multi-chamber function (more impaired RV, LV and LA function), as quantified by speckle-tracking strain assessment.

Synthesizing complex clinic data to provide tailored clinical recommendations is becoming an increasingly difficult artform for healthcare providers. This task is somewhat simplified with clinical risk calculators, but these have historically been generated through use of traditional statistical models such as logistic and linear regression together with cox proportional hazard models. These methods are advantageous in that they don't require extensive computational power but are limited by their applicability to less complex data. As we have demonstrated, unsupervised machine learning can distil complex multidimensional data into clinically important information. Interestingly, traditional markers of adverse prognostic risk, such as age, BMI or even serum haemoglobin were amongst the least relevant features with respect to cluster allocation. Moreover, STS and Rockwood Clinical Frailty scores were statistically similar between clusters. This does not by any stretch discredit the indisputable importance of these risk tools, but our observation that cluster allocation improved predictive modelling beyond these risk tools lends weight to the argument that there is a wealth of untapped insight hidden within bystander clinical and imaging data.

Echocardiography forms the cornerstone of surveillance recommendations for patients who have undergone aortic valve replacement (8). To improve the granularity of cardiac phenotyping, we included multi-chamber strain data in our input features. Strain imaging more accurately quantifies left ventricular dysfunction and is a better prognostic discriminator, particularly in patients with aortic stenosis, but is not featured in contemporary risk calculators (18). In feature ranking, it was interesting to note the LV GLS was ranked 2nd highest importance in predicting cluster allocation, behind LVOT VTI. The increased sensitivity of LV GLS in detecting sub-clinical ventricular dysfunction in patients with aortic stenosis, as compared with LVEF, may partly explain this (18). Additionally, the dominant influence of the LVOT VTI in feature rankings is particularly interesting given the prognostic importance of transvalvular flow state in patients with aortic stenosis (19). Moreover, we were also interested to observe that some important parameters of prosthesis function were discordant between clusters. Patients in the prognostically less favourable cluster had smaller indexed prosthesis areas, lower dimensionless indices, together *lower* mean gradients (albeit with borderline significance) in the setting of poorer cardiac flow state, as quantified by indexed stroke volume, together with increased prevalence of patient prosthesis mismatch. Several other groups have performed cluster-based phenotyping of patients with severe AS, albeit for pre-procedural risk stratification, with similarly interesting results (14, 15). A distinguishing factor is our inclusion of multi-chamber strain data and the inclusion of post-TAVR imaging data to quantify the state of the unloaded heart.

We have demonstrated that simple cluster-based phenotyping of comprehensive patient data post-TAVR can identify a high-risk cohort, at over 6-fold increased risk of 12-month mortality. This observation naturally raises the question of whether enhanced post-procedural follow-up and surveillance imaging, or targeted medical therapy, might improve outcomes for the high-risk cohort. Further research addressing this question is required. Additionally, there is a need for prospective randomised comparisons between conventional vs. ML-directed risk stratification to investigate superiority with respect to both procedural and post-procedural outcomes, including re-hospitalisation, major cardiac events, and death. Our observations help lay the foundation for future research integrating clustering-based insights into supervised learning models. Such models could explicitly link cluster-related features to clinical outcomes, potentially facilitating the development of a more practical risk prediction tool. We anticipate that the uptake of automated, real-time, ML-driven clinical workflows could significantly improve the speed and accuracy of healthcare recommendations for patients and guide surveillance recommendations.

## 5 Limitations

One of the limitations of k-means clustering is the use of only continuous data. Although categorical variables can be forced to be continuous with the use of techniques such as one-hot encoding, we elected not to perform this as it can introduce significant imbalance in variable weighting. Caution must be taken when simplifying feature importance with regression modelling, as cluster allocation is determined by the complex interplay between all input variables for the specific cohort studied. Our cohort was relatively small with relatively short follow-up which may limit generalisability, though the use of 37 variables for 200 patients yields over 7,000 data points and prognostically significant phenogroups were clearly identifiable. Unmeasured confounding variables may influence our findings, as is common in observational studies. We recognize that residual confounding cannot be entirely excluded. Future studies leveraging large datasets from multi-centre trials and registries, may provide additional insights to address this, and may permit more robust identification of phenogroups. Our study also includes retrospectively collected information, which can introduce bias, however our population includes a real-world, contemporary TAVR population. One of the strengths of our analysis is the use of multi-modality imaging (including CT) to comprehensively capture geometric and functional cardiac data.

## 6 Conclusion

k-means clustering identified two prognostically distinct phenogroups within our population of patients who had undergone TAVR and outperformed traditional risk-prediction tools. The prognostically less favourable cluster, associated with a 6-fold increased risk of 12-month mortality, demonstrated more advanced cardiac remodelling and poorer indices of cardiac function. With the likely future widespread application of AI, the knowledge of which patient phenogroups are at low clinical risk post-TAVR, and which are at higher risk, should be of significant clinical value and will allow physicians to triage patients to differing frequency and intensity of post-TAVR follow-up and surveillance. Ultimately, it is hoped that the individualized tailoring of follow-up and post-procedural care based on advanced risk stratification tools can lead to improved patient outcomes and reduced cost to healthcare systems globally.

## Data Availability

The datasets presented in this article are not readily available due to institutional requirements. Requests to access the datasets should be directed to the corresponding author.
