# Supplementary material for: Machine learning cluster analysis identifies increased 12-month mortality risk in transcatheter aortic valve replacement recipients
Source: Front Cardiovasc Med. 2025 Feb 5;12:1444658. doi: 10.3389/fcvm.2025.1444658 (PMC11836646; doi:10.3389/fcvm.2025.1444658)
Supplement: Supplementary file 1 [file Datasheet1.docx]

Supplementary Material

# Supplementary Tables

Table S1. Features excluded due to significant collinearity with one or more included features.

| Feature |
| --- |
| LV End Systolic Diameter (LVESD)  LV Mass  LV end diastolic volume (LVEDV)  LV end systolic volume (LVESV)  LV end systolic volume index (LVESVi)  LVOT cross sectional area (echo)  Stroke volume (SV)  LA Volume  LVOT peak velocity  AV peak velocity  AV mean velocity  AV peak gradient  AV velocity time integral (VTI)  LV longitudinal strain (LS) 2-chamber  LV LS 3-chamber  LV LS 4-chamber  Annulus minimum diameter (CT)  Annulus maximum diameter (CT) |

Table S2: TRIPOD Checklist

| **Section/Topic** | **Item** | **Checklist Item** | **Page** |
| --- | --- | --- | --- |
| **Title and abstract** | | | |
| Title | 1 | Identify the study as developing and/or validating a multivariable prediction model, the target population, and the outcome to be predicted. | 1-4 |
| Abstract | 2 | Provide a summary of objectives, study design, setting, participants, sample size, predictors, outcome, statistical analysis, results, and conclusions. | 1-4 |
| **Introduction** | | | |
| Background and objectives | 3a | - Explain the medical context (including whether diagnostic or prognostic) and rationale for developing or validating the multivariable prediction model, including references to existing models. | 3 |
|  | 3b | - Specify the objectives, including whether the study describes the development or validation of the model or both. | 3 |
| **Methods** | | | |
| Source of data | 4a | - Describe the study design or source of data (e.g., randomized trial, cohort, or registry data), separately for the development and validation data sets, if applicable. | 4 |
|  | 4b | - Specify the key study dates, including start of accrual; end of accrual; and, if applicable, end of follow-up. | 4 |
| Participants | 5a | - Specify key elements of the study setting (e.g., primary care, secondary care, general population) including number and location of centres. | 4 |
|  | 5b | - Describe eligibility criteria for participants. | 4 |
|  | 5c | - Give details of treatments received, if relevant. | NA |
| Outcome | 6a | - Clearly define the outcome that is predicted by the prediction model, including how and when assessed. | 6 |
|  | 6b | - Report any actions to blind assessment of the outcome to be predicted. | NA |
| Predictors | 7a | - Clearly define all predictors used in developing or validating the multivariable prediction model, including how and when they were measured. | 5 |
|  | 7b | - Report any actions to blind assessment of predictors for the outcome and other predictors. | NA |
| Sample size | 8 | - Explain how the study size was arrived at. | 6 |
| Missing data | 9 | - Describe how missing data were handled (e.g., complete-case analysis, single imputation, multiple imputation) with details of any imputation method. | 6 |
| Statistical analysis methods | 10a | - Describe how predictors were handled in the analyses. | 9 |
|  | 10b | - Specify type of model, all model-building procedures (including any predictor selection), and method for internal validation. | - 6,9 |
|  | 10d | - Specify all measures used to assess model performance and, if relevant, to compare multiple models. | 6,9 |
| Risk groups | 11 | Provide details on how risk groups were created, if done. | 7 |
| **Results** | | | |
| Participants | 13a | - Describe the flow of participants through the study, including the number of participants with and without the outcome and, if applicable, a summary of the follow-up time. A diagram may be helpful. | 7,16 |
|  | 13b | - Describe the characteristics of the participants (basic demographics, clinical features, available predictors), including the number of participants with missing data for predictors and outcome. | 7,16 |
| Model development | 14a | - Specify the number of participants and outcome events in each analysis. | - 9,10 |
|  | 14b | - If done, report the unadjusted association between each candidate predictor and outcome. | - NA |
| Model specification | 15a | - Present the full prediction model to allow predictions for individuals (i.e., all regression coefficients, and model intercept or baseline survival at a given time point). | - Upon request |
|  | 15b | - Explain how to the use the prediction model. | - 12-15 |
| Model performance | 16 | - Report performance measures (with CIs) for the prediction model. | 9,10 |
| **Discussion** | | | |
| Limitations | 18 | Discuss any limitations of the study (such as nonrepresentative sample, few events per predictor, missing data). | 14 |
| Interpretation | 19b | - Give an overall interpretation of the results, considering objectives, limitations, and results from similar studies, and other relevant evidence. | - 12-15 |
| Implications | 20 | Discuss the potential clinical use of the model and implications for future research. | 12-15 |
| **Other information** | | | |
| Supplementary information | 21 | Provide information about the availability of supplementary resources, such as study protocol, Web calculator, and data sets. | 7 |
| Funding | 22 | Give the source of funding and the role of the funders for the present study. | 1 |

# Supplementary Figures

Figure S1: Pearson’s correlation heatmap of input variables. Strength of correlation is represented by color gradient.

Figure S2: Plot of silhouette scores for different numbers of possible clusters (k). Two clusters yielded the highest score, indicated by the dashed line.


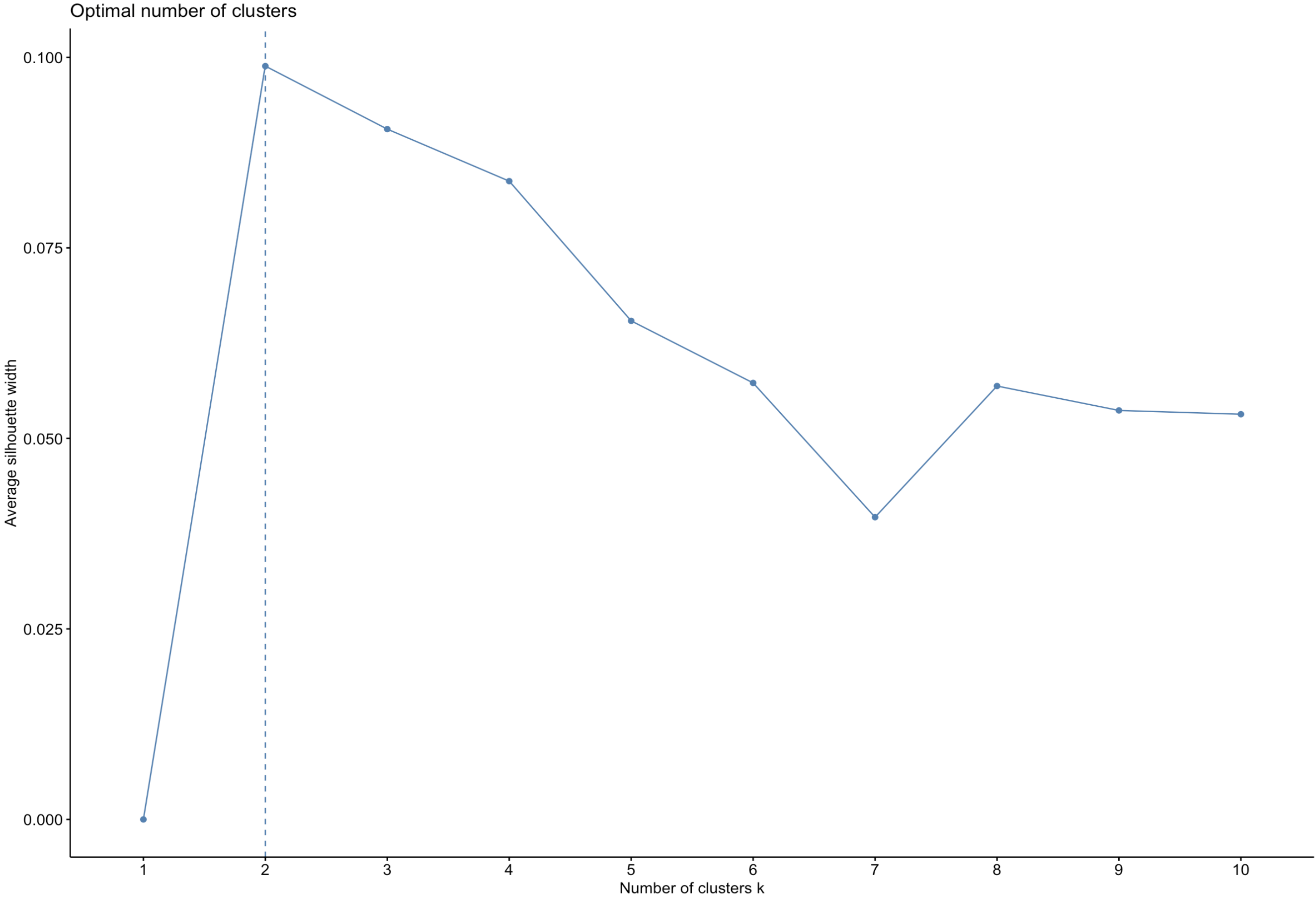


Figure S3: ROC Curve of the logistic regression model. Hyperparameter tuning identified a penalty of 1 and mixture of 10% as optimal. The logistic regression model demonstrated an accuracy of 86% in cluster prediction with an ROC-AUC of 1.0.


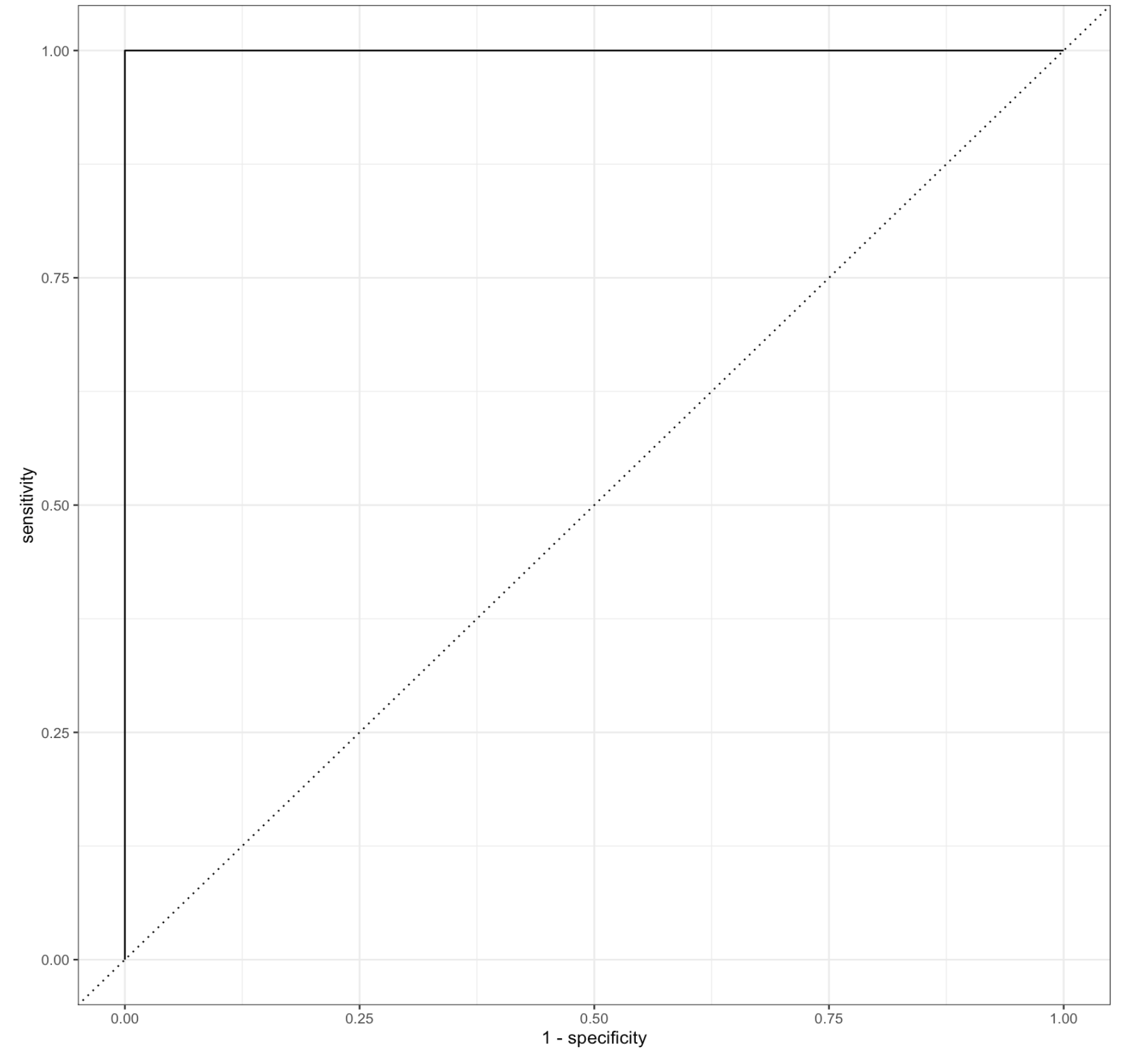


Figure S4: Feature rankings from logistic regression modelling were similar to those revealed through F scores.
